# Recognition of the microbial metabolite *p*-cresol in autism spectrum disorder: systematic review and meta-analysis

**DOI:** 10.3389/fnmol.2025.1576388

**Published:** 2025-07-02

**Authors:** María Isabel Serrano-Tomás, Paulina Contreras-Romero, Mara Parellada, Javier Chaves-Cordero, Javier Zamora, Martha Hengst, Patricia Pozo, Rosa del Campo, Sheyla Guzmán-Salas

**Affiliations:** ^1^Servicio de Microbiología, Hospital Universitario Ramón y Cajal, and Instituto Ramón y Cajal de Investigación Sanitaria (IRYCIS), Madrid, Spain; ^2^Departamento de Ciencias Farmacéuticas, Universidad Católica del Norte, Antofagasta, Chile; ^3^Servicio de Psiquiatría del Niño y del Adolescente, Facultad de Medicina, Centro de Investigación en Red en Salud Mental (CIBERSAM), Instituto de Investigación Sanitaria Gregorio Marañón (IiSGM), Instituto de Psiquiatría y Salud Mental, Hospital General Universitario Gregorio Marañón, Universidad Complutense, Madrid, Spain; ^4^Servicio de Medicina Preventiva y Salud Pública, Hospital Universitario Ramón y Cajal, Madrid, Spain; ^5^Unidad de Bioestadística, Hospital Universitario Ramón y Cajal and Instituto Ramón y Cajal de Investigación Sanitaria and Centro de Investigación Biomédica en Red de Epidemiología y Salud Pública (CIBERESP), Madrid, Spain; ^6^CIBER de Enfermedades Infecciosas (CIBERINFEC), Instituto de Salud Carlos III, Madrid, Spain; ^7^Universidad Alfonso X El Sabio, Villanueva de la Cañada, Spain

**Keywords:** autism spectrum disorder, *p*-cresol, metabolites, microbiome, gut-brain axis

## Abstract

**Introduction:**

In recent years, research has focused on the gut-brain axis and its microbial metabolites as potential etiological or physiopathological agents of autism spectrum disorders (ASDs). Elevated levels of the organic compound *para*-cresol (*p*-cresol) have been reported in various populations of children with ASD, suggesting that it could be validated as a possible ASD biomarker related to microbiota. The aim of this study was to perform a systematic review of *p*-cresol in ASD along with a meta-analysis to elucidate the scientific evidence of its potential as a biomarker.

**Methods:**

A search was performed in the PubMed, Web of Science and Scopus databases in May 2024. The Axis critical appraisal tool was used to evaluate the methodological quality of the studies included in the review. Three independent reviewers examined the identified records and performed data extraction.

**Results:**

The systematic review yielded 15 articles, of which only 6 were ultimately used for the meta-analysis. Urinary *p*-cresol levels were significantly higher in those with ASD than in healthy controls, whereas no significant differences were observed in feces.

**Conclusion:**

This meta-analysis validates that in ASD an increased level of *p*-cresol is detected in urine, which could represent a marker of microbiota evolution assessment in the pathogenesis of the disease. However, further research is needed to determine whether there is a causal relationship between the role of this metabolite and the pathophysiology of ASD and to validate its clinical utility.

## Introduction

Autism spectrum disorders (ASDs) are a group of early-onset neurodevelopmental disorder is characterized by impairments in social interaction and communication, with restricted and stereotyped behaviors (Lord et al., 2020). Worldwide, 1/100 children are currently diagnosed with ASD, with global rates affected by geographic, ethnic, and socio-economic factors, with a male/female ratio of 4.2/1 (Roman-Urrestarazu et al., 2021; Zeidan et al., 2022). However, the actual prevalence is likely underestimated (Salari et al., 2022; Roman-Urrestarazu et al., 2021; Zeidan et al., 2022). A proper diagnosis is essential for early treatment of the individual and contributes at the collective level to promoting public policies regarding autism.

The considerable presence of comorbidities present in ASD complicates its diagnosis, especially at early ages. Currently, the diagnosis is made by assessing symptomatology, including socio-affective and stereotyped, restrictive and repetitive behaviors, and following the criteria already established, such as the Diagnostic and Statistical Manual of Mental Disorders, 5th Edition or International Classification of Diseases 11th Revision scales, and if necessary, with the support of testing. Currently, the search for accurate markers remains a priority for improving early diagnosis.

The comorbidities associated with ASD are not only at the level of the CNS, such as intellectual disability, Attention-Deficit/Hyperactivity Disorder (ADHD) or developmental delay. Additionally, gastrointestinal comorbidities have been reported in 30–50% of individuals with ASD, the most common being constipation, bloating, abdominal pain and diarrhea (Buie and Margolis, 2024). A contribution to ASD of gut microbiota and their metabolites has been proposed (Penzol et al., 2019; Madra et al., 2020; Leader et al., 2022). The aromatic metabolite *para*-cresol (4-methylphenol or C_7_H_8_O; *p*-cresol) is a product of exclusive bacterial synthesis from the tyrosine amino acid which is excreted in the urine and feces. It can be found as p-cresylsulfate, p-cresylglucuronide and free p-cresol approximately in a 95:4:1 ratio (ref). *p-*cresol inhibits the dopamine *β*-hydroxylase enzyme, which converts the neurotransmitter dopamine to norepinephrine (Tran and Mohajeri, 2021); in a murine model, its availability affected systemic dopamine levels (Pascucci et al., 2020; Bermudez-Martin et al., 2021). In those with ASD, altered levels of dopamine and serotonin have been reported, with the highest percentage of serotonin being synthesized in the gut (MacFabe, 2012). Also, *p*-cresol has been widely studied *in vitro* for its impact on the central nervous system (CNS), given that it can cross the blood–brain barrier (Guzmán-Salas et al., 2022). At least 55 bacterial species have been identified as *p*-cresol producers or some of its intermediate metabolites (Zheng et al., 2021), including the class *Clostridia*, which is significantly incremented in ASD (Ho et al., 2020).

The aim of this work was to identify evidence of a relationship between *p*-cresol concentration and ASD by assessing its importance as an unspecific marker of a pro-inflammatory status known to be related to ASD outcomes during certain developmental periods (2 to 7 years of age). No meta-analysis prior to this study had been found.

## Methods

### Search strategy

The search was conducted over the period from January 2011 to May 2024, using the PubMed, Web of Science and Scopus databases. The following Medical Subject Headings (MeSH) and keyword combinations were used: (“p-cresol” OR “para-cresol” OR “4-cresol” OR “4-methylphenol” OR “p-cresylsulphate” OR “pCS” OR “p-cresylglucuronide” OR “pCG” OR “p-cresylglucoronate”) AND (“autism” OR “autistic” OR “autism spectrum disorder” OR “ASD”) NOT (review or systematic review). PubMed database option search was “all fields.” Web of Science database option search was “theme” in all databases. Scopus database option search was “Title, Abstract, Keywords.” This study is reported according to Preferred Reporting Items for Systematic Reviews and Meta-Analyses (PRISMA) guidelines (Moher et al., 2009).

### Selection criteria of the studies

**Figure 1:**
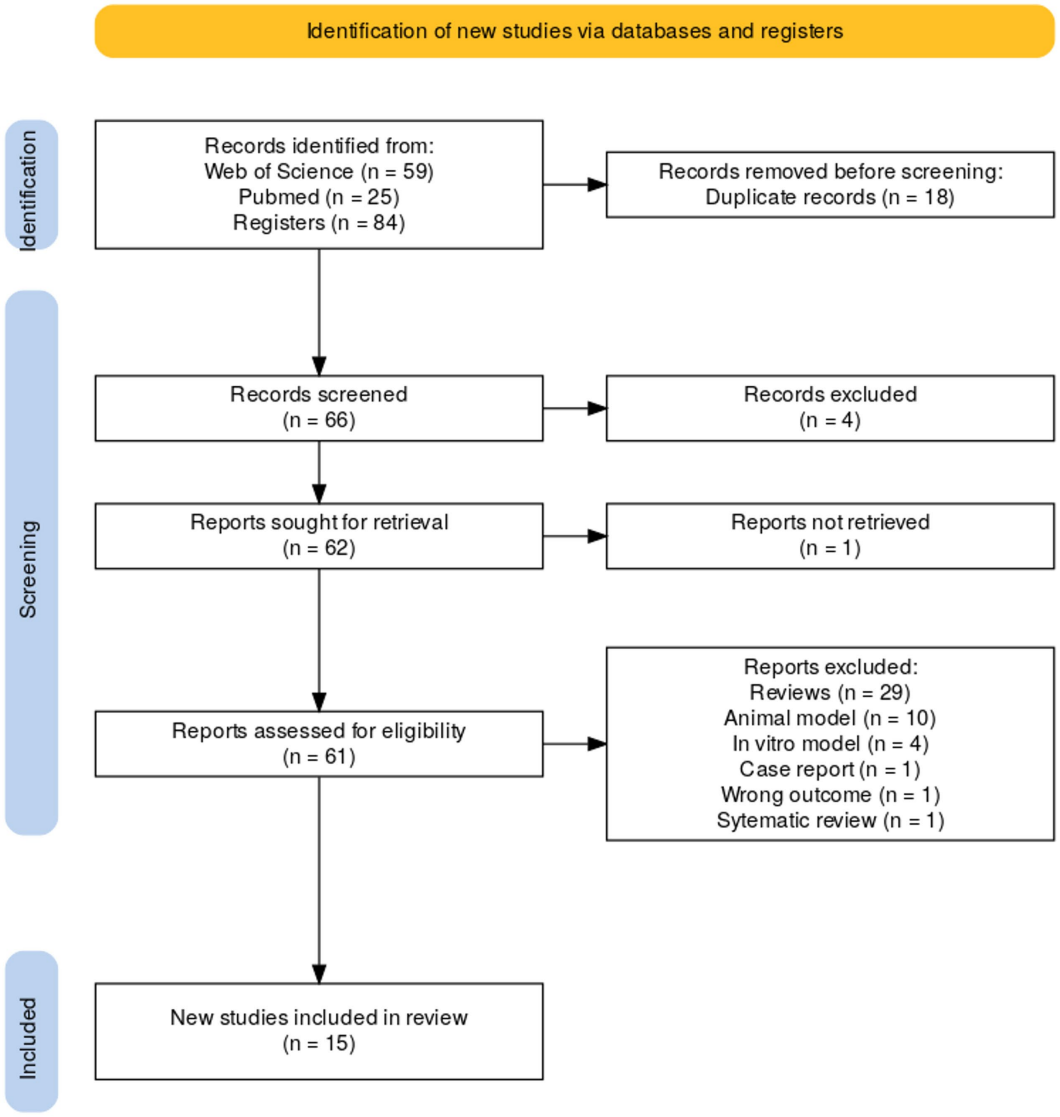
PRISMA flow diagram of study selection process.

Initially, the search yielded 556 results (Figure 1). For both the review and meta-analysis, the inclusion criteria were children under 10 years of age, diagnosed with ASD, with or without gastrointestinal disorders, we focused on articles that had conducted a metabolomics analysis of patients with ASD that detected total *p*-cresol in biological samples (urine or feces). All articles that quantified this metabolite were selected for meta-analysis. Therefore, reports on animal and *in vitro* models, case reports, reviews, and systematic reviews were excluded. Lastly, only manuscripts published in English and with full text available were included.

### Data extraction and assessment of study quality

Two independent researchers read each abstract separately and excluded or included articles retrieved from the search as appropriate. If there was disagreement in the selection of articles, a third researcher resolved this discrepancy based on the content of the abstract and eligibility criteria. Once articles met the inclusion criteria, they were downloaded in RIS or PubMed format and entered Rayyan™ software (Johnson and Phillips, 2018). The detailed methodology for article processing and exclusion/inclusion is shown in the PRISMA flowchart (Figure 1).

In the selected articles, we included a protocol for extracting information about country, study design, publication year, ASD and neurotypical (NT) control group sample sizes, the number of women in the ASD and NT groups, mean age of the ASD and NT participants, presence or absence of gastrointestinal symptomatology, ASD diagnostic criteria, biological sample type and quantification, and *p*-cresol detection method (Table 1).

**Table 1:** Characteristics of the studies included in the meta-analysis.

Reference	Country	Study design	No. of subjects (Male/female)	Age range in years	Sibling controls	Unrelated controls	Autism diagnosis	Comorbidity
Altieri et al. (2011)	Italy	Case–control	59 (44/15)	2–18		59	DSM-IV	None
Wang et al. (2012)	China	NA	23 (21/2)	33–17	22	9	NA	GI disorder
De Angelis et al. (2013)	Italy	NA	10	4–10	10		DSM-IV	None
Gabriele et al. (2014)	Italy	Case–control	33 (29/4)	4–16		33	DSM-IV	None
Gabriele et al. (2016)	Italy	Case–control	53 (44/9)	1–20		59	DSM-IV	None
Kang et al. (2018)	USA	Cohort	23 (22/1)	6–14		21	ATEC/PDD-BI	GI disorder
Kang et al. (2020)	USA	Open label trial	18	7–16		20	ADI-R	GI symptoms
Turriziani et al. (2022)	Italy	Prospective-transversal observational	21 (18/3)	2–8			DSM-IV	None

### Quality assessment

The methodological quality of the included studies was assessed employing an Axis analysis (Page et al., 2021) with the Microsoft Excel 365® program. This program serves as an instrument for critical reading and evaluation of cross-sectional epidemiological studies, consisting of a list of 20 questions that act as indicators for appropriately classifying the quality of evidence. Each question was individually applied to each evaluated article and answered based on the following criteria: yes (meets), no (does not meet), not measurable (not detailed), or not applicable (N/A). Lastly, the quality of the articles was determined based on the final score obtained after subjecting them to this questionnaire. Articles were considered of high quality if they scored >15 points, medium quality for scores 12–15 points, and low quality for scores <12 points.

### Meta-analysis and heterogeneity assessment

Random-effects meta-analyses were performed with Review Manager (Version 5.1). Mean differences (MDs) and their 95% confidence intervals (CIs) were estimated, with a positive MD indicating no differences between groups. The Q-statistic was derived, and the chi-squared test was conducted for testing the interstudy heterogeneity in effect sizes. A *p*-value <0.10 indicated significant heterogeneity. Heterogeneity in effect sizes between studies was explored using I-square (I^2^) statistics. An I^2^ value <25, >25, >50, or >75% indicates low, moderate, substantial, or considerable heterogeneity, respectively. Forest plots were created to visualize the point estimates of the study effects and their 95% confidence interval (CI), using the Stata 18.0 software. Given that no randomized controlled trials were included in this review and meta-analysis, no risk of bias assessment was performed.

## Results

### Characteristics of the included studies

The search yielded 556 articles, 170 of which were published in PubMed, 197 in Web of Science, and 189 in Scopus. A total of 281 were eliminated as duplicates; after reviewing the title/abstract, 221 were excluded. Of the 54 remaining records, 18 were excluded because they were studies conducted in animal models; 2 were *in-vitro* models; 6 were reviews; 1 was wrong population; 10 were wrong study design; 1 was a case report; and 1 had the wrong outcome. Ultimately, a total of 15 articles was selected (Altieri et al., 2011; Wang et al., 2012; De Angelis et al., 2013; Gabriele et al., 2014; Gabriele et al., 2016; Kang et al., 2018; Kang et al., 2020; Mussap et al., 2020; Gevi et al., 2020; Timperio et al., 2022; Turriziani et al., 2022; Piras et al., 2022; Daneberga et al., 2022; Vernocchi et al., 2023; Osredkar et al., 2023; Figure 1). According to the corresponding author addresses, the articles were published in 5 different countries: Italy, France, China, USA, and Slovenia, between 2011 and 2024, and only 3 studies assessed functional gastrointestinal disorders.

### Urinary levels of *p*-cresol

Statistically higher concentrations of *p*-cresol and its derivative metabolite *p*-cresol sulfate (PCS) and *p-*cresol *glucuronate* (PCG) were detected in urine of ASD participants compared with controls (Altieri et al., 2011; Gabriele et al., 2014; Gabriele et al., 2016; Mussap et al., 2020; Gevi et al., 2020; Turriziani et al., 2022; Piras et al., 2022; Daneberga et al., 2022). Similarly, Osredkar et al., detect high levels of PCS.

The first study of our selection was published in 2011 by Altieri et al. in children with severe ASD, in which the urinary total *p*-cresol concentration was 123.5 ± 12.8 μg/mL, whereas in NT children it was 91.2 ± 8.7 μg/mL. The authors emphasized that *p*-cresol levels were associated with gender and age, with higher levels in the female and younger children (134.1 ± 20.1 μg/mL vs. 70.3 ± 6.7 μg/mL); while no significant differences between ASD and controls were detected in children older than 8 years (Altieri et al., 2011).

Subsequently, in 2014, Gabriele et al. conducted a study in pediatric patients in France, aiming to replicate the initial findings in Italy by Altieri et al. (2011). The authors extended the study by further measuring the three components of urinary PCS, PCG, and free *p*-cresol. The results showed increased levels of *p*-cresol in ASD (98.8 ± 17.3 μg/mL) *vs* controls (52.0 ± 7.8 μg/mL), highlighting the percentages corresponding to PCS, PCG, and free *p*-cresol (94.99, 4.93, and 0.08%, respectively, in ASD vs. 94.87, 5.05, and 0.08% in controls). In addition, a direct relationship between *p*-cresol and p-cresol sulfate levels and clinical observations, such as delayed age at walking onset, as well as the presence of motor stereotypes and compulsive/repetitive swallowing behaviors, was observed.

Afterward, two independent studies (Gabriele et al., 2016; Mussap et al., 2020) suggested that the increase in *p*-cresol is a direct consequence of the decrease of intestinal motility together with alterations in the gut microbiota and an excessive activation of the immune system. Gabriele et al. (2016) compared the *p*-cresol levels in relation to the gastrointestinal symptoms, detecting higher values for constipation (218.2 ± 40.8 μg/mL) than for regular stools (97.9 ± 20.5 μg/mL) or diarrhea (88.6 ± 27.1 μg/mL). Mussap et al. confirmed that *p*-cresol concentration was directly proportional to ASD core symptom severity. Later, Daneberga et al. correlated higher levels of *p*-cresol with a lower *Bacteroidetes/Firmicutes* ratio, without reaching statistical significance (*p* = 0.059).

Gevi et al. (2020) correlated urinary *p*-cresol with dopamine levels in children exposed to a tyrosine-deficient diet, finding a possible justification for its mechanism of action. Accordingly, elevated dopamine levels have been linked to ASD symptoms such as stereotyped behaviors (Lewis et al., 2007; Bromberg-Martin et al., 2010). Another interesting finding was the detection of elevated vitamin C levels in ASD, which is an essential cofactor for the inactivation of dopamine *β*-hydroxylase, which catalyzes the conversion of dopamine into norepinephrine (Southan et al., 1990).

Although the seven previous studies reported elevated urinary *p*-cresol levels in relation to ASD symptomatology, Turriziani et al. did not find this association. Thus, they suggest that *p*-cresol does not contribute to behavioral changes, and accordingly, they observed no significant differences in the various ASD symptom scales after bowel mobilization in autistic children with chronic constipation.

**Figure 2:**
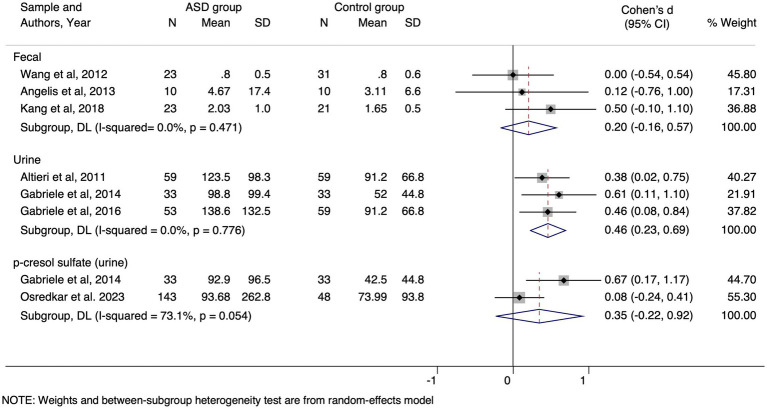
Forest plot comparing p-cresol concentration in feces and urine, and PCS in urine from subjects with ASD and healthy controls. SD, standard deviation; I2, I-square.

The meta-analysis showed that urinary *p*-cresol levels were statistically higher in ASD than in healthy controls, with great homogeneity in the results of the three included articles. In contrast, statistical significance was not reached for urinary PCS. There was heterogeneity among the studies, although the trend in the two selected articles was also for higher levels in ASD than in controls (Figure 2).

### Fecal levels of *p*-cresol

Four studies reported higher levels of *p*-cresol in the feces of children with ASD with respect to NT controls (De Angelis et al., 2013; Kang et al., 2018; Kang et al., 2020; Vernocchi et al., 2023) whereas differences were not detected in 1 study (Wang et al., 2012). The studies that detected a positive association with *p*-cresol also reported that tyramine O-sulfate, a metabolite with a similar chemical structure to 4-ethylphenyl sulfate and PCS, was under-represented in ASD compared with healthy controls. Later in 2020, in an open-label trial, Kang et al. described the beneficial effects of fecal microbiota transfer, which reduced the elevated levels of PCS, 4-hydroxy phenylacetate, and indole in feces, and modified the abundance of *Prevotella*, *Bifidobacterium*, and *Desulfovibrio*.

*Desulfovibrio*, a bacterium involved in sulfate reducing processes in the human gut, did not correlate with *p*-cresol levels; however, it did show a significant negative correlation with PCS, which might suggest a potential marker for those microorganisms capable of reducing sulfate. In addition, PCS and sulfate levels in feces showed a significant correlation with each other. Lastly, Vernocchi et al. describes a statistical association of *p*-cresol to the ASD subgroup without antibiotics administration. Despite these findings, although Wang et al. (2012) did not observe significant differences between ASD and the control groups, they did find differences between the 2 control groups (siblings and non-relatives) involved in the study. Meta-analyses in fecal samples were not conclusive, but again, *p*-cresol levels were consistently higher in the ASD group, although not reaching a statistically significant difference.

## Discussion

Gut-brain axis research has provided new insight into how the gut microbiota and its metabolites can impact neurodevelopmental conditions such as ASD (Korteniemi et al., 2024). The lack of early diagnostic biomarkers favors research on bacterial metabolites from gut microbiota, such as *p*-cresol, which has been found in elevated concentrations in the urine and feces of children with ASD. Here, we conducted the first meta-analysis that systematically reviewed published data and examined levels of *p*-cresol and its derivatives, measured in biological samples of patients with ASD.

An analysis of the previously published data did not provide evidence of a relevant association between the metabolite and ASD in feces. The studies included in the meta-analysis (Wang et al., 2012; De Angelis et al., 2013; Kang et al., 2018) were homogeneous, indicating consistency in effect sizes reported. This finding is further supported by the absence of significant heterogeneity among the included. Therefore, we can conclude that detecting this metabolite in feces may have limited utility.

However, urinary levels of *p*-cresol were up to 40% higher in ASD population than in the healthy NT controls (Altieri et al., 2011; Gabriele et al., 2014, 2016; Turriziani et al., 2022), affecting more evidently children between 2 and 7 years of age, a critical period for brain development (Altieri et al., 2011). Regarding the meta-analysis of urine studies (Altieri et al., 2011; Gabriele et al., 2014; Gabriele et al., 2016), our results showed a moderate-high pooled effect (*p* = 0.46) with low heterogeneity (I^2^ = 0.00%), suggesting that the detection of *p*-cresol in urine could have potential as a biomarker for ASD.

How *p*-cresol might affect the ASD condition is not clear; several studies conducted *in vitro* and *in vivo* have indicated that it could be associated with changes at the CNS level (Bermudez-Martin et al., 2021; Guzmán-Salas et al., 2022), particularly at early ages. However, it is certainly a metabolite that deserves attention as a potential biomarker of the microbiological alteration occurring in ASD.

The gut microbiome in ASD is characterized by a higher abundance of *Clostridia*, with a significant decrease of *Bifidobacterium* (Ho et al., 2020). Also, at the metabolic level, high levels of neuroactive metabolites derived (Finegold et al., 2017) from gut bacteria have been observed in a subset of children diagnosed with ASD (Korteniemi et al., 2024). Metabolomics provides strong evidence for a relationship between the core severity symptoms assessed by ADOS-2 and the urinary metabolic profile (Mussap et al., 2020).

### Limitations

Despite our exhaustive search, the meta-analysis is inherently limited by the low number of included studies. First, the study design, specificity, and sensitivity of the detection methods used in the included studies varied. The methodologies for *p*-cresol determination are diverse; however, not all of them specified the metabolites detected or provided average concentrations based on multiple measurements. Furthermore, confounders such as sex, genetics, dietary factors, medication, and lifestyle, as well as subtypes of bacterial species, might contribute to insignificant or falsified results. Second, significant heterogeneity was found between studies when the data on fecal samples were pooled. All studies were case–control studies; they should be validated in case series of individuals with suspected ASD and in children with other neurodevelopmental or chronic conditions to approach the disease or process-specificity of the marker.

## Conclusion

There appears to be sufficient evidence to incorporate the systematic detection of *p*-cresol in urine in people with ASD, given that it is an easy sample to obtain and process and can be useful in the monitoring of associated proinflammatory gastrointestinal pathology, as well as in microbiota alterations. Our work also highlights the importance of incorporating prognostic biomarkers related to the intestinal microbiota in this pathology. Another important fact is the need to standardize the process of *p*-cresol determination and to establish the limits of normality to be able to compare the results between populations.

## Data Availability

The original contributions presented in the study are included in the article/supplementary material, further inquiries can be directed to the corresponding authors.
